# Vulnerability Assessment of Farmland Groundwater Pollution around Traditional Industrial Parks Based on the Improved DRASTIC Model—A Case Study in Shifang City, Sichuan Province, China

**DOI:** 10.3390/ijerph19137600

**Published:** 2022-06-21

**Authors:** Yibo Zhang, Hao Qin, Guanping An, Tao Huang

**Affiliations:** 1 School of Emergency Management, Xihua University, Chengdu 610039, China; qinhao@stu.xhu.edu.cn; 2 Faculty of Geosciences and Environmental Engineering, Southwest Jiaotong University, Chengdu 611756, China; gpan@my.swjtu.edu.cn (G.A.); taohuang70@swjtu.edu.cn (T.H.)

**Keywords:** traditional industrial parks, groundwater, vulnerability assessment, analytic hierarchy process (AHP), the improved DRASTIC model

## Abstract

In this study, an improved DRASTIC model, including the DRASTIC-LTPD model and the AHP-DRASTIC-LTPD model, with the addition of four extra evaluation factors, including land use type (L), aquifer thickness (T’), aquifer potential (P) and pollution source distance (D’), was constructed and compared to assess the groundwater vulnerability around farmland area in Shuangsheng Industrial Park, Sichuan Province, China. From the vulnerability grading charts of the traditional DRASTIC model, the improved DRASTIC-LTPD model and the AHP-DRASTIC-LTPD model, it showed that the vulnerability presented a lower level in the western and eastern farmland areas, whereas a higher level was in the central industrial park area. This result was consistent with the actual situation where groundwater recharge by rivers, regional land use, and human activities were more active in the middle in these areas. Nevertheless, the area at the same level of vulnerability varied greatly from model to model. The vulnerability index V-level region ratio calculated by the AHP-DRASTIC-LTPD model was 0, indicating that the distribution of vulnerability was smoother without the appearance of extremely good or poor conditions. From the present study, it was revealed that the AHP-DRASTIC-LTPD model could effectively reflect the impact of human activities and dilution on groundwater vulnerability. The adopted AHP method was also of high accuracy to empower the evaluation index leading to a more reliable evaluation results of regional groundwater vulnerability in comparison with the other two models. Therefore, this research could be employed as a reference for the evaluation of groundwater pollution around other similar unplanned industrial parks.

## 1. Introduction

The concept of “groundwater vulnerability” was put forward in the 1960s. The U.S. National Scientific Research Council proposed groundwater vulnerability as the tendency and possibility of pollutants reaching a particular location above the uppermost aquifer. Inherent vulnerability, also known as essential vulnerability, is the manifestation of the groundwater system’s ability to adapt to changes in the external environment [1]. It emphasizes the natural properties of regional aquifers, which are static, immutable and uncontrollable by man, including the studies on the possibility of contamination, regardless of specific pollutants [2]. The special vulnerability characterizes the pollution source caused by human activities and the influence of land resources on the natural flow field of groundwater in the process of land resource development, which is dynamic and controllable and is the embodiment of the sensitivity of groundwater when disturbed by the outside world. In addition, its size is determined by the type, scale and migration and transformation law of pollutants in the groundwater environment [3,4,5]. Most studies that investigated the inherent vulnerability of groundwater, often proposed other terminologies including “groundwater pollution-prone”, “antifouling properties”, “pollution potential”, etc., to replace “groundwater vulnerability” [6,7,8]. The overlapping index, process mathematical simulation and statistical methods are three most commonly used methods in groundwater vulnerability assessment at present. The common mathematical models developed are DRASTIC model (**D**epth to water, net **R**echarge, **A**quifer media, **S**oil media, **T**opography, **I**mpact of vadose zone, and hydraulic **C**onductivity), GOD model (**G**roundwater occurrence, **O**verlying lithology of aquifer, and **D**epth to groundwater table), AVI model (**A**quifer **V**ulnerability **I**ndex) and so on [9,10,11]. Among them, the DRASTIC model is a typical representative of groundwater vulnerability assessment, and it is the most widely applied and mature groundwater vulnerability assessment method that has been successfully applied in groundwater vulnerability assessments in the United States [12], Africa [13], the European Union [14] and other regions.

To date, many scholars have used the DRASTIC model to evaluate groundwater vulnerability from both experimental and simulation means [15,16]. Groundwater vulnerability evaluation methods include overlapping indices, process mathematical simulations, statistical methods and fuzzy mathematics. The DRASTIC model is used in present study to evaluate and map groundwater vulnerability by combining mathematical means and geographical information system (GIS), which belongs to the system parameter method in the groundwater vulnerability [14,17,18,19]. The applicability of the DRASTIC model to the assessment of groundwater vulnerability in a certain area usually consists of four requirements, i.e., pollutants on the surface, pollutants can penetrate into the ground through rainfall, pollutants can be migrated with water, and the area of the study area is greater than or equal to 0.4 km^2^ [20]. Pacheco et al. [21] analyzed the statistical model of the DRASTIC model and compared the sensitivity analysis, spielman hierarchy correlation, logical regression and the corresponding analysis on the weight of their parameters and found that the corresponding analysis was the best. Based on the characteristics of groundwater in western Jilin, Bian et al. [22] increased four evaluation factors based on the traditional DRASTIC index model, namely, groundwater extraction intensity, groundwater quality, diving evaporation intensity and land use, which constituted the MEQU-DRASTIC index model, and evaluated the environmental vulnerability of groundwater in Tongyi County. Ye et al. [23] took groundwater in the second Songhua River basin as the research object, and used the measured chromium content to verify and compare groundwater environmental vulnerability by DRASTIC method and GOD method.

It can be seen from the literature review that based on the characteristics of the DRASTIC model, the problems in the evaluation of groundwater pollution are mainly the selection of parameters and the determination of weights, etc. Therefore, the selection of parameters may vary from location to location. For example, after examining the underground water quality in Saudi Arabia, Khan et al. [24,25] found that the seawater intrusion was of significant influences on the compositions of underground water. Unfortunately, there was no research on investigating the groundwater vulnerability in southwestern China, especially in the Sichuan province. From the present study, we hope it not only provides first-hand data for evaluating the groundwater quality in southwestern China, but also establishes a more accurate model to assess the groundwater vulnerability in such area and serves as database for the future researches. Therefore, in this study, an updated DRASTIC-LTPD model, which was named as DRASTIC-LTPD (this will be explained in detail in later section), was constructed by adding four evaluation factors. The evaluation factor weights were further calculated by using analytic hierarchy method, referred to as AHP-DRASTIC-LTPD model, which was constructed to evaluate groundwater vulnerability in the traditional industrial park. The vulnerability assessment of the groundwater pollution was carried out on the farmland area surrounding Shuangsheng Industrial Park, on the verge of the Shiting River, Shifang City, Sichuan Province, China.

## 2. Study Area

**Figure 1:**
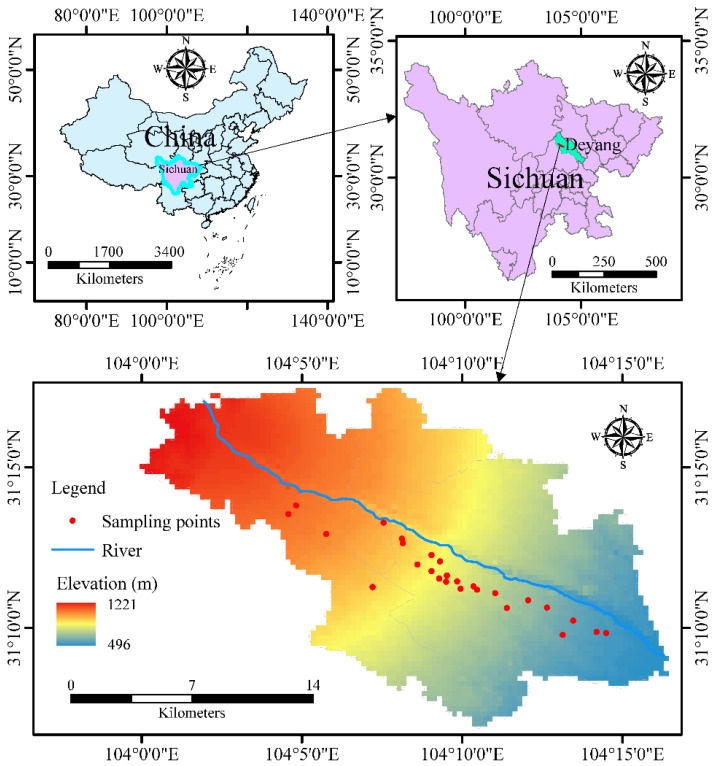
Location of the studied area.

The studied area is located in the northern part of Shifang city, Sichuan Province, as shows in Figure 1, which covers approximately 864 km^2^. The geographical coordinates are 104°09′ E and 31°10′ N, showing a trend from northwest to southeast, with an average altitude of 507 m. The region is rich in phosphate rock, raw coal, limestone and other mineral resources, and is an important phosphate rock production base in China. Moreover, the study area has a humid subtropical climate, with an annual average temperature of 16.4 °C and an annual rainfall of more than 1000 mm. The groundwater is mainly classified as pore water of the Quaternary loose rock and fissure water of cretaceous red bed sandstone mudstone. The former is mainly distributed in the quaternary gravel layer, whereas the latter is mostly buried in the fissures of the shallow weathered zone of the basement bedrock. The detailed sampling points are also marked in the diagram.

## 3. Materials and Methods

### 3.1. DRASTIC Model

Seven main influencing factors were selected in the resent study as the evaluation factors of the DRASTIC model, including groundwater depth, net recharge, aquifer media, soil media, topography, impact of vadose zone, and hydraulic conductivity, as suggested by [26]. The evaluation factor is divided into several segments (for continuous variables) or several major media types in the model. Each segment is given a score based on its relative importance within the indicator. The score is ranging from 1 to 10. Table 1 lists the evaluation factor index classification and scoring table [27,28]. Each factor is weighted according to its importance to the impact of vulnerability, with weights ranging from 1 to 5. The combined score for groundwater vulnerability is marked as *D* and calculated as follows [29]:

(1) $$D=\overset{\;7}{\underset{i=1}{\sum }}(R_{i}\times W_{i})$$

where *D* is the comprehensive index of groundwater vulnerability, dimensionless; *R_i_* represents the score value of evaluation factor *i*, dimensionless; and *W_i_* represents the weight of evaluation factor *i*, dimensionless.

### 3.2. DRASTIC Model Improvement

The traditional DRASTIC model is simple and widely used, but it is an empirical method with shortcomings existing in the application process [30]. By employing the method of first partitioning and then calculating with the research divided into different blocks, different advantages can be achieved, such as easy to calculate, easy to grasp, and quick in obtaining the results of groundwater vulnerability assessment. However, the disadvantage is that the groundwater vulnerability zoning is artificially delineated in advance, so that the differences in groundwater vulnerability indicators within different zones are alienated. The ability of spatial information contained in the map cannot be fully utilized, and the evaluation results cannot be detailed to react to the groundwater vulnerability distribution law of the study area [31,32]. The traditional DRASTIC model ignores the negative effects of single indicators, e.g., the greater the aquifer permeability coefficient is, the greater the probability of groundwater pollution is. Without considering the permeability coefficient ambassador water flow alternately accelerated dilution, that is, the stronger the dilution is, the less susceptible to groundwater pollution the water is. In addition, if the study distinguishes between valley plain and the piedmont plain, the assignment of groundwater depth can not only reflect the difference between the plain area and the mountain valley itself, or even the score which is contrary to the actual situation [30,33].

In addition, the traditional DRASTIC model adopts a classical linear weighted scoring method, and the comprehensive index obtained does not reflect the integrity of the groundwater system well. Although the external natural environment and hydrogeological conditions have been considered, the influence of human activities and pollution source characteristics were ignored. Pollution source distance factors reflect the distance of aquifers from pollution sources, and the land use category determines the size of pollution possibility, all of which have some impact on groundwater vulnerability [30,34].

Based on the above considerations, the traditional DRASTIC model is improved in the evaluation of groundwater vulnerability. After field investigation and data collection, four evaluation factors including land use type, thickness of aquifer, aquifer potential and distance of pollution sources were added to the model, and the DRASTIC-LTPD model in line with the actual situation of the study area was established. Thickness and potential of aquifer reflect the dilution effect on groundwater vulnerability, whereas land use type reflects the impact of human activities, and distance to pollution means the distance between aquifer and pollution source [28,35,36]. The index grading and scoring table of the newly added four evaluation factors are listed in Table 2.

The vulnerability composite index of the improved comprehensive evaluation model was calculated by a comprehensive scoring method, such as the DRASTIC model, the scoring value of each factor and the product of the factor weight value. Finally, the product is added and summed, and the calculation formula is as follows:

(2) $$D_{i}=\overset{n}{\underset{i=1}{\sum }}(R_{\mathrm{ij}}\times \;W_{j})$$

where *D_i_* is the evaluation point *i* of the groundwater vulnerability composite index, with no quantitative outline; *R_ij_* indicates the evaluation factor *j* of the evaluation point *i* score value, with no quantitative outline; and *W_j_* represents the weight of evaluation factor *j*, with no quantitative outline. According to the comprehensive score value, the vulnerability zoning is carried out to evaluate the groundwater vulnerability in the study area.

### 3.3. Determination of Evaluation Factors

Based on the hydrogeological conditions and on-site investigation in the study area, 11 indicator factors of the actual DRASTIC-LTPD model in the study area were determined, which will be introduced below.

#### 3.3.1. Groundwater Depth (D)

Groundwater depth reflects the distance from the surface of the pollutants through the envelope to the surface of the groundwater, determining the surface pollutants that reach the aquifer before the experience of various hydrogeochemical processes. The deeper the groundwater level is, the longer the pollutant is exposed to the envelope media, and the more pollutant reactions are physical adsorption, chemical reactions, biodegradation, etc. [37,38]. The result is that pollutants are diluted and adsorbed during the migration process, and the likelihood that harmful elements contained in pollutants will be oxidized is greater; thus, it is less likely that the groundwater will be contaminated. The more significant the decay of pollutants is, the higher vulnerability of groundwater is and vice versa. The water level buried deep in the study area is affected by the rock nature, topography and climatic conditions of the aquifers. Along the northwest-southeast line, the research area is divided into three parts: upper, middle and lower. The upper part is located in the area of Luoshui town, belonging to the Shitingjiang alluvial fan top, and the groundwater is buried deep, generally 10 to 20 m. The central part is in Luoshui town with a groundwater buried depth of 4 to 6 m, and the lower part is located at the end of the alluvial fan with a groundwater buried depth of 1 to 3 m.

#### 3.3.2. Net Recharge (R)

The net recharge is the amount of water entering the aquifer through the envelope band at a given time, usually in terms of annual net recharge. The recharge amount consists of the infiltration of atmospheric precipitation, the infiltration of canal system rivers, the infiltration of irrigation, etc., and atmospheric precipitation is the main source of groundwater recharge [39]. Net recharge has a dual impact on groundwater vulnerability. Recharge water is the main carrier of leaching and transmitting pollutants; the more water that seeps in, the more pollutants that are brought to the submersible aquifer from recharge water, and the worse the vulnerability of groundwater is. At the same time, when the recharge is large enough to dilute the pollutants, the likelihood of pollution decreases, and the vulnerability of groundwater improves [35,40]. The source of supply in the study area includes rainfall infiltration resupply and surface water infiltration resupply. The annual precipitation in the study area is 967.8 mm, whereas the annual evaporation is greater than 1000 mm, so the canals in the plain area are dense, and they form a water network area, which is another important source of groundwater recharge. Local sections of the river water recharge the groundwater, and local areas of groundwater recharge the river water or do not fill the drainage. In the Shiting River fan-top section of the river water recharge groundwater, the dry period compared to the length of the peak river supply is reduced. According to field investigations, the depth of excavation of the branch canals in the plain area is from 2 to 4 m. Except in the first year of the canal repair and channel dredging period, water is quite abundant. In the fan-top and fan-upper parts, the groundwater buried depth is more than 5 m. In the channel water supply, there is a channel water recharge groundwater situation, and the rest of the section of the channel discharges approximately two-thirds of the canal groundwater. From 15 May to the end of June each year, it is the period of the rapid rise of the water table and the peak stabilization period of the water table, after which the water table begins to decline sharply after late August. In summary, the net groundwater recharge in the study area is less than 50 mm.

#### 3.3.3. Aquifer Media (A)

Aquifers not only have the capacity to accommodate water but can also allow a considerable amount of water penetrate through, e.g., rocks that constitute aquifers containing larger loose rock particles, cracks or karst pipes. The larger the scale of the aquifer permeability is and once the pollutants enter the aquifer, the lower the chance and degree of dilution are and the higher the groundwater vulnerability is [41,42]. At the same time, some loose soil bodies must consider the impact of the grade, especially the fourth series of accumulation layers; the better the grade is, the smaller the gap in the medium is, which will increase the pollutants in the processes of transport and transfer with the aquifer medium contact time and contact area; this process reduces groundwater vulnerability [43]. According to the field investigation of the research area, as well as various geological reports and drilling data, the aquifer medium of the research area is mainly composed of sand pebbles. The upper aquifer of the area is mainly alluvial sand pebbles with layer thickness from 12 to 20 m. The middle is the mud-bearing gravel layer showing a thickness of 10 to 13 m, whereas the lower layer is 15 to 20 m thick.

#### 3.3.4. Soil Media (S)

Soil medium refers to the part of the air belt with bioactive features at the top, usually a surface weathering layer with an average thickness of 2 m or less. It obviously affects the amount of recharge that penetrates groundwater and the ability of pollutants to enter the envelope belt vertically. Soil particle size, clay mineral content, organic matter content and water content have a great influence on groundwater vulnerability [44]. The smaller the particles are, the more clay mineral content there is, the higher the organic matter content is, the higher the water content is, and the lower the vulnerability of groundwater is (and vice versa). According to the field investigation, combined with the collected data and remote sensing images, the soil in the study area was affected by climate, topography, hydrology and soil mother type, mainly sandy soil, clay soil and fine sand. The upper topsoil of the study area is powder sand and fine sand, 0.3 to 0.5 m thick, the central area near the Shitingjiang River is clay, and the rest is powder sand; and the lower part contains large areas of farmland and has sandy soil.

#### 3.3.5. Topography (T)

Topography refers to the slope of the surface, expressed as a percentage, i.e., the two-point elevation difference and its horizontal distance of the percentage. The greater the terrain slope is, the more easily pollutants migrate with surface runoff; thus, the groundwater vulnerability is better [45,46]. In most cases, when the slope value is from 0 to 2%, the terrain is flat, so the runoff carrying pollutants would stay in the area for a long time, and the water flow easily penetrates the aquifer, making the groundwater in the area highly vulnerable. When the value exceeds 18%, rainfall and surface runoff are rapidly lost to other areas, water flows have difficulty penetrating aquifers, and groundwater pollution is very low. According to hydrogeological data, the terrain of the whole area from north to west to southeast gradually slows down, and the slope change range is from 4.1 to 6.1.

#### 3.3.6. Impact of Vadose Zone (I)

The type of media in the envelope zone determines the decay characteristics of the soil medium to the pollutant between the soil layer and the aquifer, which controls the permeable path and seepage length and affects the attenuation of the contaminant and the time of contact with the medium. The rockiness of the encapsulation belt is the main factor affecting the migration and accumulation of pollutants to aquifers; the finer the rock particles are, the higher viscosity of the particles is, the worse their permeability is, the stronger the adsorption purification capacity is, and the better the vulnerability of groundwater is [47,48]. The clay layer has a strong protective effect on groundwater, and it more easily intercepts, transforms or accumulates pollutants than do the other media. With increased thickness of the clay layer, the longer it takes for pollutants to reach the aquifer, and the greater the chance the contamination will be diluted or degraded. Thus, the groundwater is less vulnerable. According to the field investigation and collected data, the upper envelope media of the study area is fine sand and medium fine sand, and the middle is powder clay, and the lower part is powdered sand.

#### 3.3.7. Hydraulic Conductivity (C)

The hydraulic conductivity reflects the permeability and hydraulic transmission performance of the aquifer medium. In anisotropic media, it is defined as the unit flow per unit of hydraulic gradient in m/d. The aquifer permeability coefficient is determined by the nature of the aquifer, including the type of rock, the porosity and clearance size of the rock interior, and the size and connectivity of karst pipes. Under certain hydraulic gradients, the permeability coefficient determines the flow rate of groundwater and the migration rate of pollutants after entering the aquifer, which affect the time of contact between groundwater and rocks around aquifers. The greater the permeability coefficient is, the faster the pollutant can leave the aquifer. Additionally, the corresponding range of contaminated water flow affects the rate of diffusion and can affect the vulnerability of groundwater. The water conductivity coefficient of the upper part of the study area is less than 200 m^2^/d; the permeability coefficient is 20 to 25 m/d; the penetration coefficient in the central region is 13 to 17 m/d; and the penetration coefficient in the lower part is 5 to 10 m/d.

#### 3.3.8. Land Use Type (L)

Human activities are an important factor that aggravates the environmental degradation of groundwater, and land use is a factor that has a great influence on groundwater in human activities. Different types of land use, precipitation infiltration on groundwater recharge process and into groundwater pollutant types, but their migration processes are not the same. Land use types are an important factor that reflect the impact of human activities on groundwater vulnerability [49]. The influence of land use on groundwater can be summarized into three aspects [50]: cultivated land, urban construction and industrial land. Fertilizer in cultivated land contains a large amount of nitrogen, phosphorus, ammonia and other elements and will cause the groundwater near the cultivated land to be higher than the abovementioned element contents. Urban construction will have an adverse effect on the infiltration of rainfall and the evaporation of groundwater. The discharge of industrial pollutants from industrial land is a serious threat to the groundwater environment. According to the field investigation and literature review, combined with the actual land use situation of the research area, the types of land use in the research area mainly include industrial land, scientific and educational land, residential land, commercial land, transportation land, forest land, grassland, cultivated land and 8 other types. Taking into account the large number of industrial parks in the research area, industrial land will be further refined into high-tech and advanced manufacturing land and general manufacturing land.

#### 3.3.9. Aquifer Thickness (T’)

The thickness of the aquifer reflects the size of groundwater storage space. The higher thickness is, the more groundwater that can be stored, the stronger the dilution capacity of groundwater is, and the better vulnerability of groundwater is. The aquifer in the study area is mainly composed of sand pebbles. The upper aquifer is mainly alluvial sand pebbles (12 to 20 m thick). The middle is the mud-containing pebble layer with thickness ranging from 10 to 13 m, and the lower layer is 15 to 20 m thick.

#### 3.3.10. Aquifer Potential (P)

Aquifer potential refers to a certain depth of drop, a certain caliber under the single well water output. The aquifer potential reflects the water storage, permeability and water conductivity of aquifers [51]. Generally, the greater aquifer potential is, the stronger the water conduction capacity of the aquifer is, the faster migration and diffusion of pollutants in the aquifer are. Under such circumstances, the concentration of pollutants is more easily diluted, and the groundwater is less susceptible to pollute [42]. In summary, the higher aquifer potentials are, the lower the vulnerability of groundwater is. The water-richness of the upper aquifer in the study area is poor, and the water output of a single-hole is generally 100 to 500 m^3^/d; the water-rich water of the central aquifer is medium, and the water content of a single-hole is generally 500 to 1000 m^3^/d; the water-rich water of the lower aquifer is better with the water content of a single-hole water generally from 1000 to 3000 m^3^/d and the discharge point spring flow from 20 to 60 L/s.

#### 3.3.11. Pollution Source Distance (D’)

**Figure 2:**
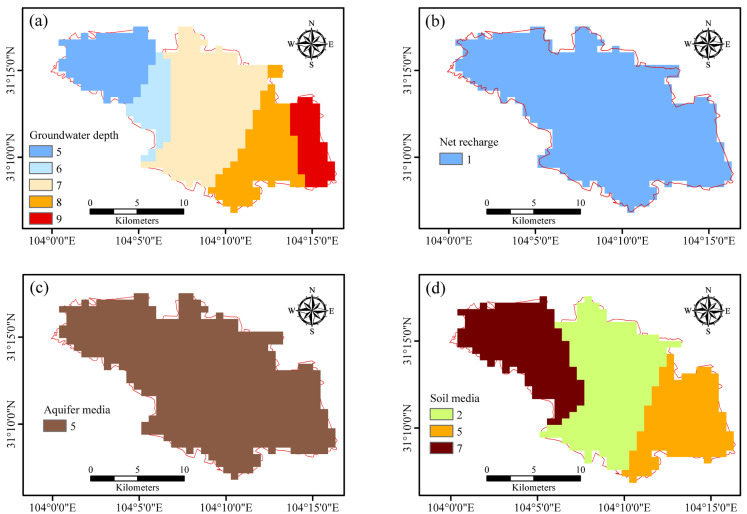

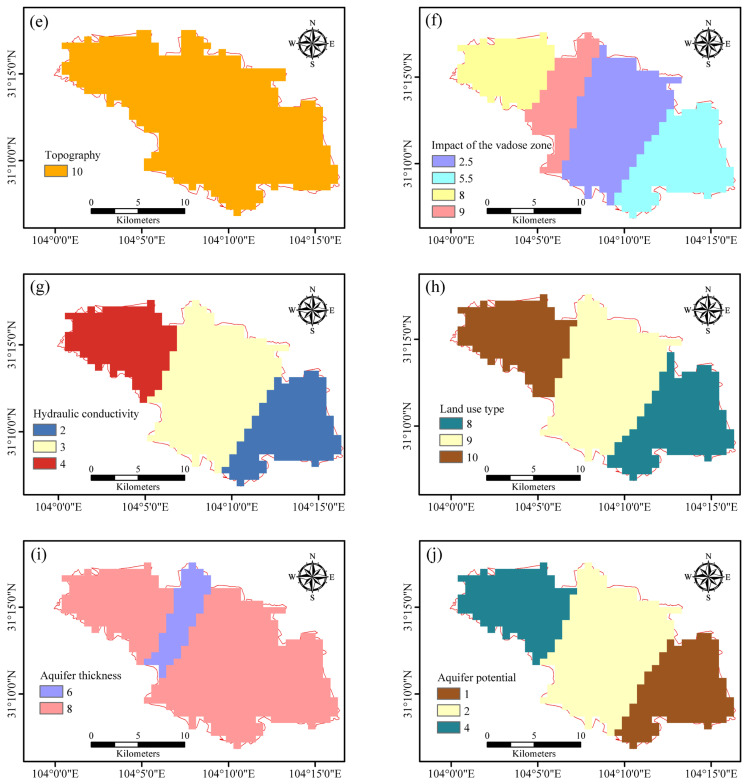

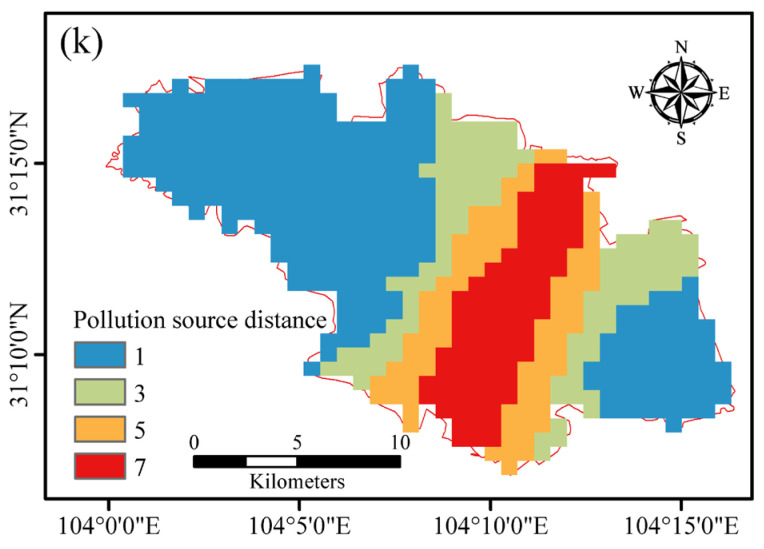
Scores of sample point evaluation factors, groundwater depth (**a**), net recharge (**b**), aquifer media (**c**), soil media (**d**), topography (**e**), impact of the vadose zone (**f**), hydraulic conductivity (**g**), land use type (**h**), aquifer thickness (**i**), aquifer potential (**j**) and pollution source distance (**k**).

The distance from the pollution source reflects the distance between the aquifer and the source of pollution. The location of the unplanned industrial park in the studied area was used as the source of pollution. The distance between each sample point and the industrial park was the distance from the source of pollution. The calculated data regarding different evaluation factors was imposed on the map and shown in Figure 2.

## 4. Results and Discussion

### 4.1. Determination of Index Weight

(1) Comprehensive scoring assignment

The selection of factor weights in the DRASTIC model is determined by measuring the importance of comparing the vulnerability of each indicator to groundwater. The traditional DRASTIC model gives weight to the index within a 1–5 circumference according to the influence of a single factor on groundwater vulnerability. The higher the weight value is, the greater impact of the indicator on groundwater vulnerability is, the greater weight value given to the indicator with the least impact on groundwater vulnerability is. The most influential indicator is given at a weight value of 5. According to expert experience, the DRASTIC model has two common weight index systems used to assess the extent of the influence of 7 evaluation factors on groundwater vulnerability. As stated previously, the general DRASTIC model is applicable to groundwater vulnerability assessment under normal conditions; however, it ignores the impact of human activities on groundwater vulnerability. This evaluation method belongs to the category of a special vulnerability assessment, commonly referred to as the pesticide vulnerability index, which is more applicable to groundwater vulnerability assessment in areas affected by agricultural activities.

Based on the particularity of the agricultural activity area, the four evaluation factors of land use type (L), aquifer thickness (T’), aquifer water potential (P) and pollution source distance (D’) were added to form the DRASTIC-LTPD model. The comprehensive scoring method needs to reassign the weight value of each evaluation factor in light of the actual situation in the studied area. Table 3 lists the weight values for the general DRASTIC model, the pesticide DRASTIC model, and the DRASTIC-LTPD model. For the weight value of soil type, 2 is used in the general DRASTIC evaluation model, and this study area is a combination of agricultural area and industrial park. Additionally, soil type has a strong influence on groundwater system, so the weight value in this study is 4. The studied area is located in the plain area, the terrain slope influence is small, and the weight assignment in the general DRASTIC evaluation model is used. The media and hydraulic conduction coefficients of the envelope belt are valued by the pesticide DRASTIC model. Because a higher the land use type leads to a greater the influence on groundwater vulnerability, in the DRASTIC-LTPD model, the weight assignment is 5; the aquifer thickness weight assignment is 2; the aquifer potential weight assignment is 2; and the pollution source distance weight assignment is 4.

(2) Improvement with AHP and determination of weight

The analytic hierarchy process (AHP) is a decision-making method in which elements that are always relevant to decision-making are broken down into goals, guidelines, scenarios, and so on [52]. Compared with the comprehensive scoring method, the hierarchical analysis method takes the research object as a system and makes the decision according to decomposition, comparative judgment and a comprehensive way of thinking. The idea of the system is not to cut off the influence of various factors on the results. The degree of influence of each factor on the results is quantified and very clear [53]. Therefore, compared with other traditional scoring methods, hierarchical analysis can better reflect the integrity of the groundwater system. For the conventional DRASTIC model, because the weight of each index is artificially determined, it does not change with the actual situation of the groundwater environment in the studied area, which affects the objective evaluation results. Using hierarchical analysis to determine the weight, that is, considering the subjective judgment, can also use the secondary structure of the transmission class to express the complex relationship of the evaluation object, layer-by-layer evaluation analysis, and finally obtain the lowest level relative to the highest level of importance of the weight [54,55].

Using the AHP method to determine the weight of DRASTIC-LTPD evaluation indicators, the research problem is divided into the target layer of solving the problem, the constraint layer of taking measures and the program layer of the implementation plan. Target layer A is the weight and constraint layer B is the hydrogeological data of 11 indicators, with groundwater depth (D), net recharge (R), aquifer media (A), soil media (S), topography (T), impact of vadose zone (I), hydraulic conduction (C), land use type (L), aquifer thickness (T’), aquifer potential (P), pollution source distance (D’). The DRASTIC-LTPD model assigns the greatest weight values to 5, the lowest values to 1, and the weights to 36, according to the traditional weight assignment method.

The construction of the judgment matrix needs to be carried out by comparing the relative importance of the factors, whereas the principle of comparison is based on the scale of relative importance, as shown in Table 4.

Each layer of factors is used to compare the judgment between each other and on each factor relative essential *a_ij_* listed the judgment matrix *A*, that is, to build the judgment matrix:

(3) $$A={\left(a_{ij}\right)}_{n\times n}=\left[\begin{array}{ccc} a_{11} & \cdots & a_{1n}\\ \vdots & & \vdots \\ a_{n1} & \cdots & a_{nn} \end{array}\right]$$

The judgment matrix is based on the relative importance of the 11 factors in the groundwater vulnerability assessment, as shown in Table 5.

In the process of calculating the weight value of the matrix, it is also necessary to test the consistency of the matrix, the purpose of which is to determine whether the matrix is reasonably available, to average the geometries of the row vectors of the matrix, and then to normalize and obtain the weight value of each factor.

The calculation process is as follows:

1. Calculate the product *M_i_* of each row element of the judgment matrix, as shown in Equation (4):

(4) $$M_{i}=\prod_{j=1}^{m}u_{ij},(i,j=1,2,\cdots ,m)$$

2. Calculate the *m*-th root *W_i_* of *m*, as shown in Equation (5):

(5) $$W_{i}=M_{i}^{\frac{1}{m}}$$

3. Normalize the vector = [*W*_1_, *W*_2_, …, *W_m_*]^T^ to obtain the weight value of the factor in the second row, as shown in Equation (6):

(6) $$W_{i}=W_{i}/\sum_{j=1}^{m}W_{j}$$

4. Calculate the maximum eigenvalue of the judgment matrix λ_max_:

(7) $$\lambda_{\max}=\sum_{i=1}^{m}\frac{{\left(PW\right)}_{i}}{nW_{i}}=\frac{1}{n}\sum_{i=1}^{m}\frac{{\left(PW\right)}_{i}}{W_{i}}$$

where (*PW*)*_i_* is the first element of the vector *PW:*

(8) $$PW=\left[\begin{array}{c} {\left(PW\right)}_{1}\\ {\left(PW\right)}_{2}\\ \vdots \\ {\left(PW\right)}_{m} \end{array}\right]=\left[\begin{array}{cccc} \mu_{11} & \mu_{12} & \cdots & \mu_{1m}\\ \mu_{21} & \mu_{22} & \cdots & \mu_{2m}\\ \vdots & \vdots & \vdots & \vdots \\ \mu_{m1} & \mu_{m2} & \cdots & \mu_{mm} \end{array}\right]\left[\begin{array}{c} W_{1}\\ W_{2}\\ \vdots \\ W_{m} \end{array}\right]$$

The resulting feature vector is the desired weight, and the evaluation factor weight is calculated as shown in Table 6.

To test its rationality, it is necessary to carry out a consistency test on the judgment matrix, and the calculation formula is as follows:

(9) CR=CI/RI

(10) $$CI=\frac{1}{m-1}(\lambda_{\max}-m)$$

In the formula, λ_max_ is the maximum characteristic root of the matrix, *m* is the matrix order, and in this study, *m* is 11. *CR* is the random consistency ratio of the judgment matrix, *CI* is the general consistency index of the judgment matrix, and *RI* is the average random consistency index of the judgment matrix. The value of the *RI* is related to the order of the matrix, e.g., when it is the 11th order matrix, the *RI* is 1.52.

When *CR* < 0.10, it is considered that the judgment matrix has satisfactory consistency, which indicates that the weight distribution of evaluation factors is reasonable. By calculating the *CI* value of 0.0577, the *CR* value of 0.0379, and *CR* < 0.10, the judgment matrix has satisfactory consistency, so the results can be adopted.

### 4.2. Analysis of Evaluation Results

Three different evaluation methods were used to evaluate groundwater vulnerability in the studied area, and the sample evaluation factor score and the weight factor under the three evaluation models were brought into Formula (2). The antifouling composite index of the DRASTIC model, DRASTIC-LTPD model and AHP-DRASTIC-LTPD model was then calculated. The available vulnerability *D_i_* values was calculated separately for these three models, and it is not possible to compare the judgments clearly because of the different ranges of the three comprehensive scoring values. In addition, groundwater vulnerability grading criteria are graded according to the traditional DRASTIC model, and it is not possible to directly compare the antifouling composite index of the improved DRASTIC-LTPD model and the AHP-DRASTIC-LTPD model. Therefore, a standardized method was used to convert the grading standard and the antifouling composite index. The standardization process is shown below:

(1) Standardization of *Di*

According to Formula (11), to convert the value to 0 to 100, the conversion results are shown in Table 7.

(11) $$D_{s}=\frac{D_{i}-D_{\min}}{D_{\max}-D_{\min}}\times 100$$

where *D_s_* is the standardized *D_i_* value, *D*_max_ is the maximum *D_i_* value, and *D*_min_ is the minimum *D_i_* value.

(2) Vulnerability classification

**Figure 3:**
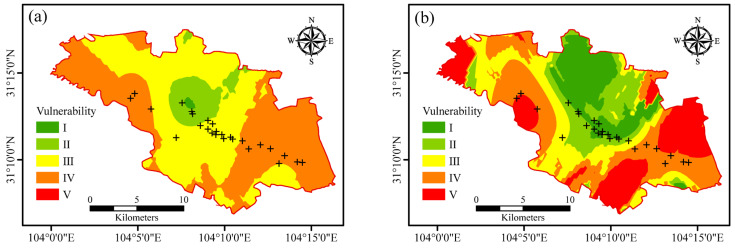

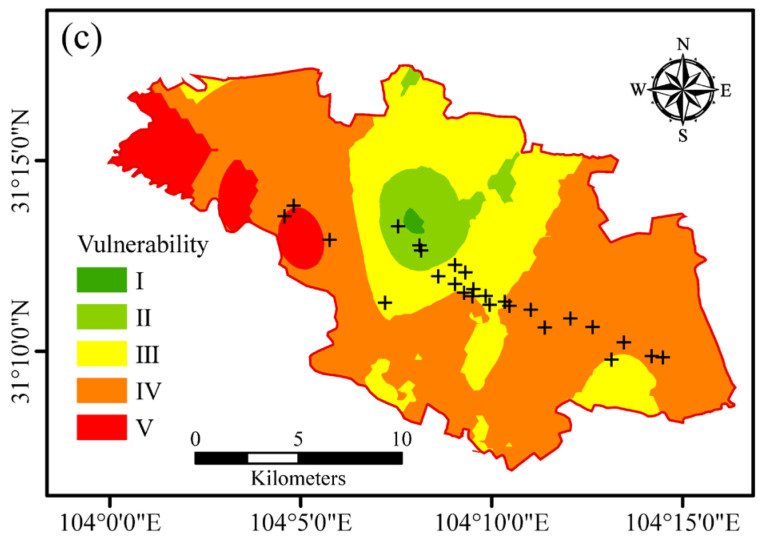
Groundwater vulnerability classification of different evaluation models: (**a**) traditional DRASTIC model, (**b**) DRASTIC-LTPD model, and (**c**) AHP-DRASTIC-LTPD model. Note that the + symbol indicates the sampling location.

According to the scoring of groundwater vulnerability in the study area, the regional score of each index was obtained by using ArcGIS spatial analysis and other functions. According to the standardized groundwater vulnerability classification criteria, vulnerability is divided into five levels: I, II, III, IV and V. Figure 3 shows the vulnerability classification maps of the traditional DRASTIC model, the improved DRASTIC-LTPD model and the AHP-DRASTIC-LTPD model. The spatial trends of the vulnerability index obtained by the three models showed that the vulnerability of the farmland areas in the west and east was poor, whereas the vulnerability of the central industrial park area was better, which was consistent with the actual situation, as the groundwater recharge by rivers, the regional land use, and the human activities are mainly concentrated in the middle of the studied area.

The area of the same level of vulnerability varied greatly from model to model, and ArcGIS was used to count the area of vulnerability classification in three models, as shown in Table 8. Comparing the area of vulnerability area at all levels of the three different evaluation models, the area of vulnerability areas at all levels calculated by the three evaluation models is quite different. The traditional DRASTIC model shows that the area of vulnerability areas at all levels was more widely distributed in comparison with other two models. The areas with vulnerability of I and II are 61.53 km^2^ and 78.93 km^2^, respectively, accounting for 16.58% and 21.27%, respectively. The largest vulnerable area is level IV, with an area of 101.6 km^2^. The proportion is 27.38%. The vulnerability index calculated by the improved DRASTIC-LTPD model and the AHP-DRASTIC-LTPD model does not have a level I vulnerability partition, indicating that the land use type (L), aquifer thickness (T), aquifer water potential (P), pollution source distance (D), and four evaluation factors had a significant effect on the evaluated model. In addition, the vulnerability index V-level region ratio calculated by the AHP-DRASTIC-LTPD model was 0, indicating that the distribution of vulnerability was smoother and did not appear extremely good or poor through the improved computational model. The AHP-DRASTIC-LTPD model reflected the impact of human activities and dilution on groundwater vulnerability by increasing the land use type, aquifer thickness and aquifer potential index. Therefore, this method used the AHP method to empower the evaluation index, making the evaluation results of regional groundwater vulnerability more reliable than the results from the other two models.

## 5. Conclusions

In view of the shortcomings of the traditional DRASTIC model and considering the influence of human activities according to the actual situation of the research area, apart from seven commonly used factors to reflect natural conditions, four additional evaluation factors: land use type, aquifer thickness, aquifer water potential, pollution source distance, were considered, with new model including DRASTIC-LTPD and AHP-DRASTIC-LTPD model proposed. The conclusions made by the present study is shown below:

(1) By comparing the vulnerability grading charts of the traditional DRASTIC model, the improved DRASTIC-LTPD model and the AHP-DRASTIC-LTPD model, the spatial change trend of the vulnerability index obtained by the three models showed that the vulnerability of the farmland areas in the west and east was poor, whereas the vulnerability of the industrial park area was better, which was consistent with the actual situation that groundwater recharge by rivers, regional land use, and human activities were concentrated in the middle of the research area.
(2) The area difference of the same level of vulnerability in different models was rather significant, whereas the vulnerability index V-level regional proportion calculated by the AHP-DRASTIC-LTPD model was 0, indicating that the distribution of vulnerability was smoother and that there was no extremely good or poor situation through the improved computational model.
(3) The AHP-DRASTIC-LTPD model reflected the impacts of human activities and dilution on groundwater vulnerability by increasing land use type, aquifer thickness and aquifer potential index and used the AHP method to empower the evaluation index, making the evaluation results of regional groundwater vulnerability more reliable than the other two models, which was considered to be the most effective model for vulnerability assessment in the present study.

## Figures and Tables

**Table 1:** Details of DRASTIC model quantitative parameters.

Ranking	Groundwater Depth (m)	Net Recharge (mm)	Aquifer Media	Soil Media	Topography (%)	Impact of Vadose Zone	Hydraulic Conductivity (m/d)
10	(0, 1]	(0, 10]	Limestone	Thin layer or missing	(0, 2]	Pebble gravel	>150
9	(1, 2]	(10, 15]	lava	Gravel	(2, 4]	Coarse sand	(100, 150]
8	(2, 4]	(15, 20]	Karst limestone	Medium sand and coarse sand	(4, 7]	Medium sand	(80, 100]
7	(4, 7]	(20, 25]	Massive limestone	Silt and fine sand	(7, 9]	fine sand	(60, 80]
6	(7, 10]	(25, 30]	Weathered metamorphic rock	Expansive or cohesive clay	(9, 11]	Silty fine sand	(45, 60]
5	(10, 15]	(30, 35]	Sand gravel	Sandy loam	(11, 13]	Silt	(30, 45]
4	(15, 20]	(35, 40]	Medium coarse sand	loam	(13, 15]	Silt	(20, 30]
3	(20, 30]	(40, 50]	fine sand	Silty loam	(15, 17]	Silt	(10, 20]
2	(30, 40]	(50, 60]	Sub sand	clay loam	(17, 18]	Silty clay	(5, 10]
1	>40	>60	Silty fine sand	Non expansive and contractile clay	>18	Clay	(0, 5]

**Table 2:** Ranking and scoring table of new additional evaluation factors.

Ranking	Aquifer Thickness/m	Land Use Type	Aquifer Potential/(m^3^·d^−1^)	Pollution Source Distance/(m)
10	(0, 10]	Flower bed	(0, 5]	(0, 50]
9	(10, 15]	General manufacturing land	(5, 10]	(50, 100]
8	(15, 20]	farmland	(10, 20]	(100, 200]
7	(20, 25]	Residential land	(20, 50]	(200, 400]
6	(25, 30]	Land for high-tech and advanced manufacturing	(50, 100]	(400, 600]
5	(30, 35]	Science and education land	(100, 200]	(600, 800]
4	(35, 40]	Cultivated land	(200, 400]	(800, 1000]
3	(40, 50]	Grassland	(400, 800]	(1000, 2000]
2	(50, 60]	forest	(800, 1000]	(2000, 5000]
1	>60	Bare stone zone	>1000	>5000

**Table 3:** Weight values of traditional indicator systems.

Evaluation Factor	Weight Value		
	General DRASTIC Model	Pesticide DRASTIC Model	DRASTIC-LTPD Model
Groundwater depth (D)	5	5	5
Net recharge (R)	4	4	4
Aquifer media (A)	3	3	3
Soil media (S)	2	5	4
Topography (T)	1	3	1
Impact of vadose zone (I)	5	4	4
Hydraulic conductivity (C)	3	2	2
Land use type (L)	/	/	5
Aquifer thickness (T’)	/	/	2
Aquifer potential (P)	/	/	2
Pollution source distance (D’)	/	/	4

**Table 4:** Relative importance statistics of AHP.

Ratio Correlation between A and B	Extremely Important	Very Important	Important	Slightly Important	Equally Important	Slightly Minor	Secondary	Very Secondary	Extremely Minor
Evaluation value	9	7	5	3	1	1/3	1/5	1/7	1/9
remarks	The values 8, 6, 4, 2, 1/2, 1, 1/4, 1/6 and 1/8 are the above intermediate values								

**Table 5:** Judgment matrix of AHP.

	(D)	(R)	(A)	(S)	(T)	(I)	(C)	(L)	(T’)	(P)	(D’)
(D)	1	2	5	4	9	3	7	2	6	7	4
(R)	1/2	1	3	1	7	1	5	1/3	5	5	1
(A)	1/5	1/3	1	1/3	5	1/3	3	1/5	3	4	1/3
(S)	1/4	1	3	1	7	1	5	1/2	6	5	1
(T)	1/9	1/7	1/5	1/7	1	1/7	1/4	1/9	1/3	1/3	1/7
(I)	1/3	1	3	1	7	1	5	1/2	5	3	1
(C)	1/7	1/5	1/3	1/5	4	1/5	1	1/4	1	1	1/5
(L)	1/2	3	5	2	9	2	4	1	6	6	3
(T’)	1/6	1/5	1/3	1/6	3	1/5	1	1/6	1	1	1/5
(P)	1/7	1/5	1/4	1/5	3	1/3	1	1/6	1	1	1/5
(D’)	1/4	1	3	1	7	1	5	1/3	5	5	1

**Table 6:** Factor weight values determined by AHP.

Evaluation Factor	(D)	(R)	(A)	(S)	(T)	(I)	(C)	(L)	(T’)	(P)	(D’)
Weight	2.920	1.274	0.621	1.269	0.157	1.210	0.320	2.190	0.293	0.300	1.212

**Table 7:** Grading standards for groundwater vulnerability.

Vulnerability Classification	I	II	III	IV	V
Evaluate	good	preferably	secondary	Poor	difference
Vulnerability index *Di*	≤70	70~90	90~120	120~160	≥160
Standardized value *Di*	0	0~22.2	22.2~55.6	55.6~100	100

**Table 8:** Statistics on vulnerability levels of different evaluation models.

Grade	Traditional DRASTIC Model		DRASTIC-LTPD Model		AHP-DRASTIC-LTPD Model	
	Area/km^2^	Scale/%	Area/km^2^	Scale/%	Area/km^2^	Scale/%
I	61.53	16.58	1.06	0.29	1.18	0.32
II	52.43	14.13	23.11	6.23	31.87	8.59
III	76.63	20.65	101.44	27.33	171.72	46.27
IV	101.6	27.38	217.63	58.64	166.35	44.82
V	78.93	21.27	27.88	7.51	0.00	0.00
total	371.12	100.00	371.12	100.00	371.12	100.00
